# Supplementary material for: High-risk histological subtype-related FAM83A hijacked FOXM1 transcriptional regulation to promote malignant progression in lung adenocarcinoma
Source: PeerJ. 2023 Oct 26;11:e16306. doi: 10.7717/peerj.16306 (PMC10613442; doi:10.7717/peerj.16306)
Supplement: Supplemental Information 1 [file peerj-11-16306-s001.docx]

**Supplementary Materials**

**High-risk histological subtype-related FAM83A hijacked FOXM1 transcriptional regulation to promote malignant progression in lung adenocarcinoma**

Fei Wei^1,2#^, Yan Yan^3#^, Guangjun Liu^4^, Bo Peng^1,4^, Yuanyuan Liu^5^, Qiang Chen^1,4^

1. The Clinical College of Xuzhou Medical University, Xuzhou, China
2. Department of Thoracic Surgery, Xuyi People’s Hospital, Xuyi, China
3. Department of Cardiovascular Medicine, The Affliated Hospital of Xuzhou Medical University, Xuzhou, China
4. Department of Thoracic Surgery, Xuzhou Central Hospital, Xuzhou, China
5. Department of Respiratory and Critical Care Medicine, Xuzhou Central Hospital, Xuzhou, China

# Fei Wei and Yan Yan contributed equally to this work

**Corresponding authors:**

Qiang Chen

The Clinical School of Xuzhou Medical University, Department of Thoracic Surgery, Xuzhou Central Hospital, Xuzhou, China

Yuanyuan Liu

Department of Respiratory and Critical Care Medicine, Xuzhou Central Hospital, Xuzhou, China

**Contents**

**Supplementary Figure S1:** FAM83A expression in patients with different clinicopathological information and the efficiency of sgFAM83A and the overexpression of FAM83A.

**Supplementary Figure S2:** The statistical results of colony formation assay, EdU assay, Transwell and Matrigel assay in vitro.

**Supplementary Figure S3:** FAM83A regulates cell cycle dependent on FOXM1.

**Supplementary Table 1:** Primer sets, Sequences of siRNAs and sgRNA sets

**Supplementary Table 2:** The mean expression of differentially expressed mRNA in low-risk pathological subtypes, intermediate-risk pathological subtypes, and high-risk pathological subtypes patients in TCGA-LUAD dataset.

**Supplementary Table 3:** Differentially expressed mRNA between high FAM83A expression patients (top 15%) and low FAM83A expression patients (bottom 15%) from TCGA database.

**Supplementary Table 4:** Differentially expressed mRNA between high FAM83A expression patients (top 15%) and low FAM83A expression patients (bottom 15%) from GSE30219.

**Supplementary Figure S1: FAM83A expression in patients with different clinicopathological information and the efficiency of sgFAM83A and the overexpression of FAM83A.**


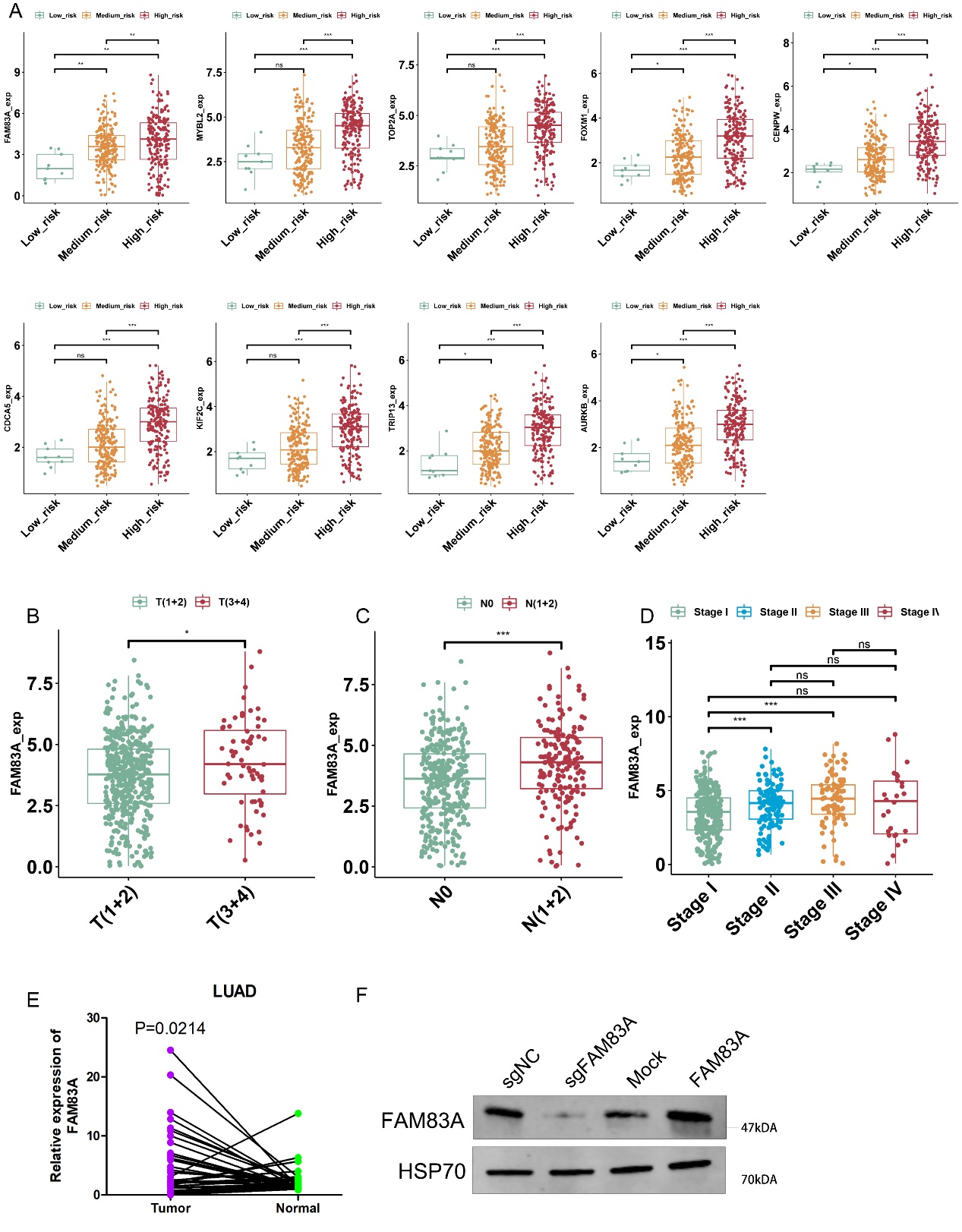


Figure S1: FAM83A expression in patients with different clinicopathological information and the efficiency of sgFAM83A and the overexpression of FAM83A. FAM83A mRNA expression distribution between pathology subtype (A) Tumor stage (B), lymph node stage (C) and TNM stage (D). (E) The mRNA expression of FAM83A in 48 pairs of LUAD tissue and matched adjacent tissue. (F) The WesternBlots revealed the expression of FAM83A after knockdown or overexpression.

**Supplementary Figure S2: The statistical results of colony formation assay, EdU assay, Transwell and Matrigel assay in vitro.**

**
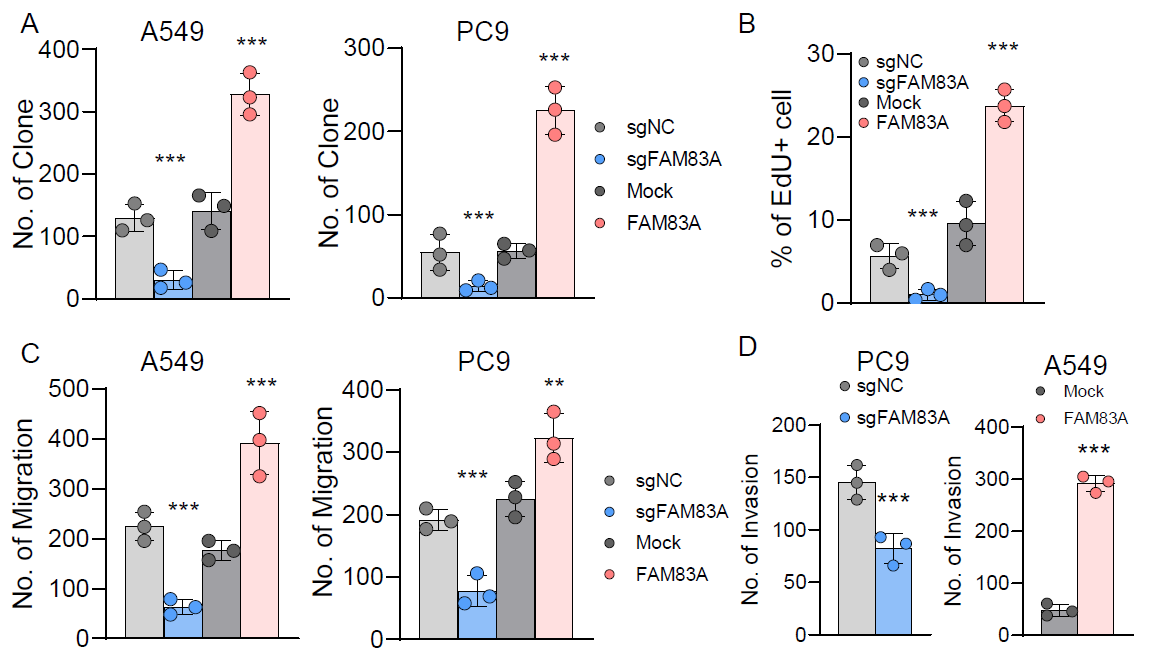
**

Figure S2: The statistical results of colony formation assay, EdU assay, Transwell and Matrigel assay in vitro. (A) Colony formation assays of A549 and PC9 cells with FAM83A knockdown or overexpression. (B) EdU assays of A549 and PC9 cells with FAM83A knockdown or overexpression. (C-D) Transwell assay and Matrigel of A549 and PC9 cells with FAM83A knockdown or overexpression. *p<0.05, **p< 0.01, ***p<0.001 (Student’s t test).

**Supplementary Figure S3: FAM83A regulates cell cycle dependent on FOXM1.**


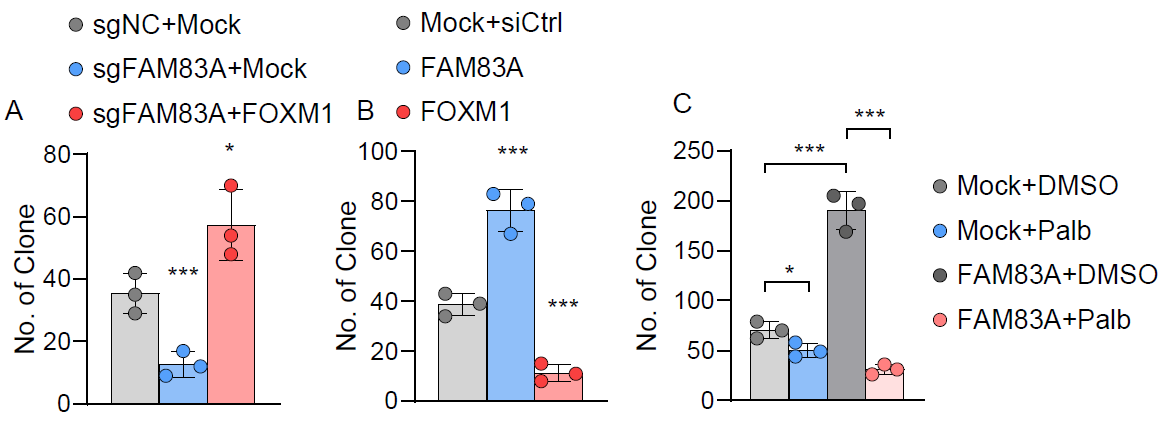


Figure S3: FAM83A regulates cell cycle dependent on FOXM1. (A-B) Colony formation revealed that cell proliferation affected by FAM83A knockdown or overexpression was reversed by cotransfection with FOXM1 knockdown or overexpression in A549 Cell line. (C) Colony formation revealed that the efficacy of Palbociclib was more significant after FAM83A overexpression in A549 cell line. *p<0.05, **p< 0.01, ***p<0.001 (Student’s t test).

**Supplementary Table 1 Primer sets, Sequences of siRNAs and sgRNA sets**

| **Primer sets** | | |
| --- | --- | --- |
| CDC6 | Forward | 5’-CAGAAAAGCATTACACAGCTGT-3’ |
|  | Reverse | 5’-GAAAATGACTGGTAGCCTGTTG-3’ |
| CDC20 | Forward | 5’-CAACTCTTTGTAACCCAGAACG-3’ |
|  | Reverse | 5’-CCTTTCCAAACACATTCGGATT-3’ |
| FOXM1 | Forward | 5’-GATCTGCGAGATTTTGGTACAC-3’ |
|  | Reverse | 5’-CTGCAGAAGAAAGAGGAGCTAT-3’ |
| CDKN3 | Forward | 5'-GCCCAGTTCAATACAAACAAGT-3' |
|  | Reverse | 5'-CAACCTGGAAGAGCACATAAAC-3' |
| CDK2 | Forward | 5’-CCAGGAGTTACTTCTATGCCTGA-3’ |
|  | Reverse | 5’-TTCATCCAGGGGAGGTACAAC-3’ |
| CDK4 | Forward | 5’-ATGGCTACCTCTCGATATGAGC-3’ |
|  | Reverse | 5’-CATTGGGGACTCTCACACTCT-3’ |
| CDC16. | Forward | 5’-TCAAAGTGCTCTATTTTGGGCA-3’ |
|  | Reverse | 5’-TTGTCCAGTTTTCGTGACCGA-3’ |
| GAPDH | Forward | 5’-GGAGCGAGATCCCTCCAAAAT-3’ |
|  | Reverse | 5’-GGCTGTTGTCATACTTCTCATGG-3’ |
| FOXM1-promoter Region 1 | Forward | 5’-gcagggagagagagagagga-3’ |
|  | Reverse | 5’-ttatcttccagggcccttgg-3‘ |
| FOXM1-promoter Region 2 | Forward | 5’-tggagtgtggtgtgagttga-3’ |
|  | Reverse | 5’-acatccaactgttctgccct-3’ |
| FOXM1-promoter Region 3 | Forward | 5’-caaatgtgggctgggcatag-3‘ |
|  | Reverse | 5’-gtgcagtggtgtgatcatgg-3‘ |
| FOXM1-promoter Region 4 | Forward | 5’-gagggagagtttggggacg-3‘ |
|  | Reverse | 5’-tgtgggaaaatggggtacga-3‘ |
| **Sequences of siRNAs** | | |
| siFOXM1-1 | gagagtgaaaacgcagattcat | |
| siFOXM1-2 | gagtgaaaacgcagattcataat | |
| siFOXM1-3 | cgcagattcataatgaaaactag | |
| **Sequences of sgRNA** | | |
| sgFAM83A | AACGAACACCCCACGCTTGT | |

**Supplementary Table 2**

|  | Low_risk | Medium_risk | High_risk |
| --- | --- | --- | --- |
| FAM83A | 2.11611 | 3.570744 | 3.944706 |
| UBE2C | 3.043474 | 3.856284 | 4.911044 |
| MMP12 | 1.66319 | 2.70543 | 3.727645 |
| BIRC5 | 1.948913 | 2.707255 | 3.667252 |
| CDC20 | 2.597576 | 3.324649 | 4.268468 |
| TPX2 | 2.442066 | 3.168673 | 4.088058 |
| MYBL2 | 2.541142 | 3.301796 | 4.199036 |
| MELK | 1.243889 | 1.892465 | 2.764526 |
| ANLN | 1.546669 | 2.320616 | 3.170854 |
| TK1 | 3.892719 | 4.369283 | 5.211939 |
| TOP2A | 2.970899 | 3.498229 | 4.333127 |
| RRM2 | 1.995265 | 2.834526 | 3.640282 |
| CTD-2510F5.4 | 1.450102 | 2.119725 | 2.916867 |
| CXCL10 | 3.169774 | 3.925463 | 4.71772 |
| FOXM1 | 1.654058 | 2.290511 | 3.083241 |
| CENPW | 2.083654 | 2.676056 | 3.465894 |
| CDCA5 | 1.624798 | 2.132678 | 2.920844 |
| KIF2C | 1.619741 | 2.207636 | 2.990877 |
| TRIP13 | 1.458802 | 2.152192 | 2.925133 |
| AURKB | 1.481733 | 2.170192 | 2.941833 |
| CCNA2 | 1.818546 | 2.381697 | 3.155358 |
| SLC2A1 | 2.841228 | 4.252606 | 4.987803 |
| CDKN3 | 1.401091 | 1.914991 | 2.689966 |
| CEP55 | 1.65811 | 2.376089 | 3.128769 |
| KIF4A | 1.298449 | 1.794866 | 2.550565 |
| DLGAP5 | 1.242569 | 1.832343 | 2.579262 |
| AQP5 | 4.30069 | 4.059804 | 2.471137 |
| C16orf89 | 8.346816 | 6.168708 | 4.556315 |
| PGC | 6.602273 | 5.226336 | 3.724602 |
| SCGB3A2 | 7.714709 | 6.535484 | 5.078562 |
| NAPSA | 9.775395 | 8.346368 | 6.903734 |
| SCGB3A1 | 7.180003 | 6.142235 | 4.736851 |
| MSLN | 4.63393 | 5.996614 | 4.710069 |
| AGR3 | 4.81879 | 5.477283 | 4.176896 |
| SFTPB | 11.89518 | 10.23596 | 8.830026 |
| CLIC6 | 4.906859 | 5.240847 | 3.93478 |
| BPIFA1 | 1.631172 | 4.689105 | 3.557979 |
| MUC5B | 1.262187 | 3.729414 | 2.586127 |
| SCGB1A1 | 5.551854 | 5.40696 | 4.165062 |
| SFTA2 | 8.426079 | 7.505625 | 6.235308 |
| SPINK1 | 3.801724 | 4.892646 | 3.716985 |
| PPP1R1B | 2.878073 | 3.437833 | 2.250771 |
| CXCL14 | 3.039464 | 4.830844 | 3.747218 |
| AQP3 | 7.460532 | 7.178253 | 6.011945 |
| PIGR | 7.305856 | 6.502608 | 5.314392 |
| CEACAM6 | 8.843895 | 8.840071 | 7.721098 |
| SFTPA2 | 9.045773 | 8.666073 | 7.549465 |
| HPGD | 3.572269 | 3.96163 | 2.903886 |
| SFTPA1 | 9.132847 | 8.38492 | 7.275854 |
| CRYM | 3.190577 | 2.727139 | 1.657264 |
| SLC44A4 | 5.697507 | 5.503866 | 4.447117 |
| SFTPC | 8.746604 | 5.281286 | 4.842013 |
| MS4A15 | 4.506748 | 2.045614 | 1.487749 |
| AGER | 6.376327 | 3.859182 | 3.505936 |
| SUSD2 | 6.560648 | 4.31269 | 3.47175 |
| GGTLC1 | 5.101158 | 3.00091 | 2.006934 |
| CYP4B1 | 5.955857 | 4.058246 | 2.997341 |
| SFTPD | 8.21768 | 6.131505 | 5.474495 |
| CYP2B7P | 6.479637 | 4.561488 | 3.606175 |
| FOLR1 | 7.902989 | 6.100646 | 5.00344 |
| WIF1 | 4.261514 | 2.252377 | 1.595308 |
| SLC26A9 | 4.65375 | 2.95491 | 1.903692 |
| ZNF385B | 3.378054 | 1.403515 | 1.16392 |
| WFDC12 | 2.377305 | 0.351612 | 0.259472 |
| SFTA1P | 5.593265 | 3.917336 | 3.148769 |
| SLC22A3 | 4.797178 | 3.268893 | 2.39651 |
| GFRA3 | 4.090492 | 2.440513 | 1.931081 |
| CES1 | 5.836745 | 3.982877 | 3.88902 |
| SLC22A31 | 6.459799 | 5.038206 | 4.095792 |
| MYBPHL | 2.990251 | 1.306638 | 0.893688 |
| AQP4 | 4.717792 | 3.106748 | 2.634681 |
| ATP13A4 | 4.019927 | 2.454071 | 1.905594 |
| CIT | 4.65837 | 3.205719 | 2.489807 |

**Supplementary Table 3**

|  | logFC | AveExpr | t | P.Value | adj.P.Val | B |
| --- | --- | --- | --- | --- | --- | --- |
| ENSG00000147689 | 7.50635 | 11.1046 | 37.08501 | 8.92E-60 | 4.85E-55 | 115.1453 |
| ENSG00000204949 | 4.894128 | 6.972431 | 30.24135 | 9.89E-52 | 2.69E-47 | 100.4877 |
| ENSG00000253258 | 2.135857 | 4.89912 | 18.5413 | 6.21E-34 | 1.12E-29 | 64.66803 |
| ENSG00000141526 | 2.229542 | 12.97373 | 11.76339 | 1.71E-20 | 2.32E-16 | 35.68738 |
| ENSG00000104140 | 3.665131 | 9.568327 | 10.76794 | 2.40E-18 | 2.60E-14 | 30.97064 |
| ENSG00000185567 | 3.287528 | 11.21342 | 10.73178 | 2.87E-18 | 2.60E-14 | 30.79804 |
| ENSG00000172927 | 4.282612 | 8.137566 | 10.13016 | 5.84E-17 | 3.97E-13 | 27.91668 |
| ENSG00000164520 | 2.060933 | 6.226748 | 9.807057 | 2.95E-16 | 1.23E-12 | 26.36418 |
| ENSG00000099812 | 2.205328 | 11.26248 | 9.673338 | 5.77E-16 | 2.09E-12 | 25.72113 |
| ENSG00000167767 | 2.405696 | 10.85349 | 9.540886 | 1.12E-15 | 3.59E-12 | 25.08406 |
| ENSG00000153294 | 2.958267 | 7.024016 | 9.452321 | 1.75E-15 | 5.05E-12 | 24.65808 |
| ENSG00000145113 | 3.490723 | 11.2439 | 9.204043 | 6.08E-15 | 1.46E-11 | 23.46429 |
| ENSG00000115641 | 2.00499 | 11.00502 | 9.200272 | 6.19E-15 | 1.46E-11 | 23.44616 |
| ENSG00000019186 | 4.505548 | 9.970767 | 9.024567 | 1.49E-14 | 3.12E-11 | 22.60206 |
| ENSG00000188910 | 3.494038 | 8.090345 | 8.799596 | 4.60E-14 | 8.33E-11 | 21.52279 |
| ENSG00000167772 | 2.815618 | 10.6265 | 8.78285 | 5.00E-14 | 8.76E-11 | 21.44254 |
| ENSG00000088002 | 2.548675 | 8.986324 | 8.702125 | 7.47E-14 | 1.23E-10 | 21.05589 |
| ENSG00000135480 | 2.265548 | 14.70304 | 8.668906 | 8.82E-14 | 1.41E-10 | 20.89689 |
| ENSG00000131746 | 3.630505 | 9.553487 | 8.551684 | 1.58E-13 | 2.20E-10 | 20.3363 |
| ENSG00000163053 | 2.597522 | 9.498587 | 8.464363 | 2.44E-13 | 3.02E-10 | 19.91929 |
| ENSG00000165474 | 2.622699 | 9.79498 | 8.440189 | 2.75E-13 | 3.14E-10 | 19.80394 |
| ENSG00000148344 | 2.667594 | 10.47062 | 8.438681 | 2.77E-13 | 3.14E-10 | 19.79675 |
| ENSG00000158825 | 3.124345 | 8.692316 | 8.423272 | 3.00E-13 | 3.32E-10 | 19.72325 |
| ENSG00000185008 | -2.35558 | 7.549151 | -8.31815 | 5.05E-13 | 4.99E-10 | 19.22228 |
| ENSG00000196167 | -2.87455 | 8.643411 | -8.22116 | 8.16E-13 | 7.78E-10 | 18.76094 |
| ENSG00000179913 | 2.551619 | 10.26009 | 8.1297 | 1.28E-12 | 1.11E-09 | 18.32664 |
| ENSG00000069011 | 2.962564 | 8.812413 | 8.069441 | 1.73E-12 | 1.47E-09 | 18.04095 |
| ENSG00000111879 | -2.07504 | 8.436753 | -8.04716 | 1.93E-12 | 1.60E-09 | 17.93541 |
| ENSG00000163993 | 4.778221 | 11.10938 | 8.045794 | 1.94E-12 | 1.60E-09 | 17.92894 |
| ENSG00000141527 | 2.087683 | 8.321273 | 8.024798 | 2.15E-12 | 1.70E-09 | 17.82954 |
| ENSG00000175592 | 2.617791 | 8.566546 | 8.010424 | 2.31E-12 | 1.79E-09 | 17.76152 |
| ENSG00000166920 | 2.475601 | 10.36101 | 7.962043 | 2.93E-12 | 2.21E-09 | 17.53274 |
| ENSG00000167644 | 2.567505 | 10.6584 | 7.936468 | 3.33E-12 | 2.44E-09 | 17.4119 |
| ENSG00000164509 | 2.052889 | 6.18453 | 7.838598 | 5.38E-12 | 3.52E-09 | 16.9502 |
| ENSG00000138759 | -2.29452 | 9.08424 | -7.80836 | 6.24E-12 | 3.90E-09 | 16.80778 |
| ENSG00000058085 | 2.474359 | 12.91894 | 7.799188 | 6.53E-12 | 4.03E-09 | 16.76462 |
| ENSG00000136002 | 2.214653 | 8.279939 | 7.72899 | 9.20E-12 | 5.32E-09 | 16.43454 |
| ENSG00000143512 | 3.053788 | 7.050732 | 7.71952 | 9.64E-12 | 5.46E-09 | 16.39006 |
| ENSG00000117394 | 2.184399 | 12.79359 | 7.676208 | 1.19E-11 | 6.35E-09 | 16.18679 |
| ENSG00000205420 | 4.83933 | 8.731524 | 7.610317 | 1.64E-11 | 7.84E-09 | 15.87805 |
| ENSG00000147257 | -2.5367 | 9.470706 | -7.59339 | 1.78E-11 | 8.29E-09 | 15.79885 |
| ENSG00000133134 | -2.03031 | 8.372743 | -7.3902 | 4.79E-11 | 1.93E-08 | 14.8514 |
| ENSG00000120875 | 2.38001 | 11.38032 | 7.374629 | 5.16E-11 | 2.05E-08 | 14.77905 |
| ENSG00000228742 | 2.29845 | 6.102007 | 7.316485 | 6.84E-11 | 2.53E-08 | 14.50931 |
| ENSG00000186832 | 3.11193 | 7.493466 | 7.255647 | 9.17E-11 | 3.26E-08 | 14.2277 |
| ENSG00000101938 | -2.48937 | 8.761439 | -7.21036 | 1.14E-10 | 3.85E-08 | 14.01852 |
| ENSG00000150893 | -2.39937 | 8.773454 | -7.0987 | 1.95E-10 | 6.05E-08 | 13.50435 |
| ENSG00000137825 | 2.441367 | 7.790584 | 7.08519 | 2.08E-10 | 6.35E-08 | 13.4423 |
| ENSG00000159708 | -2.00171 | 6.815338 | -7.07933 | 2.14E-10 | 6.49E-08 | 13.41538 |
| ENSG00000101213 | 2.03034 | 9.949825 | 6.952362 | 3.92E-10 | 1.08E-07 | 12.83422 |
| ENSG00000173267 | 2.255065 | 9.027624 | 6.886592 | 5.36E-10 | 1.37E-07 | 12.53449 |
| ENSG00000157890 | -2.02257 | 6.341735 | -6.8703 | 5.79E-10 | 1.47E-07 | 12.4604 |
| ENSG00000141338 | -2.02981 | 7.89472 | -6.86088 | 6.05E-10 | 1.53E-07 | 12.41756 |
| ENSG00000125775 | 2.247699 | 9.624011 | 6.810576 | 7.68E-10 | 1.81E-07 | 12.18925 |
| ENSG00000225329 | -2.57051 | 6.916549 | -6.78329 | 8.74E-10 | 2.00E-07 | 12.06565 |
| ENSG00000259974 | -2.69916 | 7.915863 | -6.71616 | 1.20E-09 | 2.64E-07 | 11.76229 |
| ENSG00000196878 | 2.120667 | 13.78677 | 6.715033 | 1.21E-09 | 2.64E-07 | 11.75718 |
| ENSG00000173237 | 2.309497 | 5.700888 | 6.642498 | 1.69E-09 | 3.41E-07 | 11.43061 |
| ENSG00000181143 | 3.477471 | 9.21906 | 6.6382 | 1.73E-09 | 3.43E-07 | 11.4113 |
| ENSG00000171243 | -2.14046 | 6.041066 | -6.57216 | 2.36E-09 | 4.33E-07 | 11.11516 |
| ENSG00000165197 | -2.10877 | 6.958979 | -6.50594 | 3.21E-09 | 5.48E-07 | 10.81933 |
| ENSG00000147697 | 2.131854 | 7.800102 | 6.483851 | 3.55E-09 | 5.82E-07 | 10.7209 |
| ENSG00000138271 | 2.709531 | 7.1932 | 6.480181 | 3.62E-09 | 5.83E-07 | 10.70456 |
| ENSG00000172264 | -2.44041 | 8.918493 | -6.46793 | 3.83E-09 | 6.05E-07 | 10.65002 |
| ENSG00000248323 | 2.05181 | 7.146915 | 6.448147 | 4.20E-09 | 6.55E-07 | 10.56207 |
| ENSG00000167656 | 2.924236 | 6.516329 | 6.40991 | 5.01E-09 | 7.44E-07 | 10.39236 |
| ENSG00000111700 | 2.427145 | 5.4498 | 6.317709 | 7.67E-09 | 1.03E-06 | 9.984791 |
| ENSG00000088926 | -2.03968 | 5.733738 | -6.31301 | 7.84E-09 | 1.04E-06 | 9.964095 |
| ENSG00000166923 | 2.307874 | 10.0345 | 6.24127 | 1.09E-08 | 1.31E-06 | 9.648719 |
| ENSG00000123999 | 3.036844 | 6.997895 | 6.196813 | 1.34E-08 | 1.52E-06 | 9.454045 |
| ENSG00000124466 | 2.391147 | 9.03638 | 6.171818 | 1.50E-08 | 1.66E-06 | 9.34485 |
| ENSG00000165973 | -2.77231 | 6.013793 | -6.1301 | 1.81E-08 | 1.93E-06 | 9.163024 |
| ENSG00000114248 | -2.07185 | 6.624411 | -6.12621 | 1.84E-08 | 1.95E-06 | 9.146089 |
| ENSG00000160886 | 2.719193 | 7.033162 | 6.115169 | 1.94E-08 | 2.03E-06 | 9.098075 |
| ENSG00000196616 | -3.05284 | 9.253513 | -6.10426 | 2.04E-08 | 2.09E-06 | 9.050649 |
| ENSG00000122133 | 3.179808 | 7.167518 | 5.991743 | 3.39E-08 | 3.15E-06 | 8.563867 |
| ENSG00000174564 | 2.197395 | 7.461563 | 5.938431 | 4.31E-08 | 3.79E-06 | 8.334637 |
| ENSG00000080031 | 2.234258 | 8.550661 | 5.914164 | 4.80E-08 | 4.16E-06 | 8.230608 |
| ENSG00000120211 | 2.83358 | 5.542832 | 5.867303 | 5.92E-08 | 4.92E-06 | 8.030276 |
| ENSG00000165092 | -2.01814 | 11.83125 | -5.8491 | 6.42E-08 | 5.28E-06 | 7.952637 |
| ENSG00000131910 | -2.22071 | 6.400059 | -5.8475 | 6.47E-08 | 5.30E-06 | 7.945848 |
| ENSG00000102854 | 3.203593 | 12.14594 | 5.83219 | 6.93E-08 | 5.61E-06 | 7.880653 |
| ENSG00000205426 | 2.77274 | 7.031498 | 5.788281 | 8.42E-08 | 6.56E-06 | 7.694154 |
| ENSG00000058335 | -2.24375 | 8.494245 | -5.78377 | 8.59E-08 | 6.67E-06 | 7.675014 |
| ENSG00000107984 | 2.828181 | 8.212114 | 5.771529 | 9.07E-08 | 6.97E-06 | 7.623175 |
| ENSG00000108924 | -2.05987 | 8.476055 | -5.73392 | 1.07E-07 | 7.99E-06 | 7.464178 |
| ENSG00000204019 | 2.491873 | 5.984783 | 5.732092 | 1.08E-07 | 8.04E-06 | 7.456481 |
| ENSG00000206073 | 2.093388 | 5.369028 | 5.723192 | 1.12E-07 | 8.28E-06 | 7.418935 |
| ENSG00000115457 | -2.12705 | 12.19275 | -5.72291 | 1.13E-07 | 8.28E-06 | 7.417759 |
| ENSG00000084110 | 2.366061 | 8.01958 | 5.717597 | 1.15E-07 | 8.46E-06 | 7.395348 |
| ENSG00000146013 | -2.36578 | 7.316157 | -5.71357 | 1.17E-07 | 8.52E-06 | 7.378399 |
| ENSG00000007402 | -2.38513 | 9.914613 | -5.63029 | 1.69E-07 | 1.14E-05 | 7.028783 |
| ENSG00000206075 | 2.887402 | 7.80527 | 5.569636 | 2.21E-07 | 1.39E-05 | 6.775752 |
| ENSG00000060718 | 2.384992 | 9.383943 | 5.556583 | 2.34E-07 | 1.45E-05 | 6.721483 |
| ENSG00000137440 | 2.072956 | 7.202407 | 5.543139 | 2.48E-07 | 1.52E-05 | 6.665659 |
| ENSG00000127249 | -2.37474 | 9.240388 | -5.52605 | 2.67E-07 | 1.62E-05 | 6.594809 |
| ENSG00000124664 | 2.1658 | 9.851256 | 5.521374 | 2.72E-07 | 1.64E-05 | 6.575427 |
| ENSG00000275216 | 2.421435 | 6.006757 | 5.503467 | 2.94E-07 | 1.76E-05 | 6.501329 |
| ENSG00000154975 | -2.01865 | 5.099373 | -5.49498 | 3.05E-07 | 1.81E-05 | 6.466242 |
| ENSG00000115919 | 2.304422 | 10.39871 | 5.482802 | 3.22E-07 | 1.89E-05 | 6.415971 |
| ENSG00000087128 | 2.537569 | 7.221718 | 5.48006 | 3.26E-07 | 1.90E-05 | 6.404657 |
| ENSG00000167653 | 3.014278 | 7.583637 | 5.404918 | 4.50E-07 | 2.43E-05 | 6.09578 |
| ENSG00000148702 | -2.53483 | 8.037145 | -5.32258 | 6.41E-07 | 3.22E-05 | 5.759951 |
| ENSG00000167755 | 2.667364 | 6.525789 | 5.316844 | 6.57E-07 | 3.28E-05 | 5.736644 |
| ENSG00000148346 | 2.38182 | 11.53746 | 5.31066 | 6.75E-07 | 3.34E-05 | 5.711546 |
| ENSG00000268104 | 2.16224 | 10.97999 | 5.262819 | 8.27E-07 | 3.93E-05 | 5.517928 |
| ENSG00000198183 | 3.619243 | 8.461576 | 5.244842 | 8.93E-07 | 4.15E-05 | 5.445423 |
| ENSG00000164161 | -2.11997 | 7.779827 | -5.23101 | 9.46E-07 | 4.34E-05 | 5.389732 |
| ENSG00000227471 | 2.055589 | 6.496271 | 5.199133 | 1.08E-06 | 4.80E-05 | 5.261687 |
| ENSG00000142973 | -2.74642 | 10.06526 | -5.156 | 1.30E-06 | 5.50E-05 | 5.089111 |
| ENSG00000185479 | 2.133052 | 5.971464 | 5.143535 | 1.37E-06 | 5.72E-05 | 5.039412 |
| ENSG00000116031 | -2.11636 | 6.962793 | -5.11713 | 1.53E-06 | 6.22E-05 | 4.934334 |
| ENSG00000156076 | -2.49165 | 7.925864 | -5.11681 | 1.53E-06 | 6.22E-05 | 4.933035 |
| ENSG00000057149 | 2.586163 | 6.28336 | 5.060876 | 1.93E-06 | 7.34E-05 | 4.711466 |
| ENSG00000144331 | -2.02705 | 8.26263 | -5.04227 | 2.09E-06 | 7.78E-05 | 4.63806 |
| ENSG00000124882 | 2.357082 | 7.457817 | 4.989307 | 2.60E-06 | 9.20E-05 | 4.430002 |
| ENSG00000163220 | 2.132967 | 12.57168 | 4.931265 | 3.30E-06 | 0.000111 | 4.203464 |
| ENSG00000074410 | 2.021145 | 10.26846 | 4.911731 | 3.58E-06 | 0.000119 | 4.127576 |
| ENSG00000170561 | -2.18676 | 8.854347 | -4.85112 | 4.59E-06 | 0.000142 | 3.893249 |
| ENSG00000146374 | 2.081735 | 7.907657 | 4.841549 | 4.77E-06 | 0.000147 | 3.856419 |
| ENSG00000066405 | -2.95661 | 9.254803 | -4.76429 | 6.52E-06 | 0.000188 | 3.560676 |
| ENSG00000099994 | -2.14875 | 11.29995 | -4.73628 | 7.29E-06 | 0.000205 | 3.454155 |
| ENSG00000112936 | -2.15733 | 10.57457 | -4.72901 | 7.51E-06 | 0.000209 | 3.426572 |
| ENSG00000188505 | 2.058373 | 7.293326 | 4.704354 | 8.29E-06 | 0.000227 | 3.333247 |
| ENSG00000196611 | 2.193914 | 9.964349 | 4.665411 | 9.68E-06 | 0.000253 | 3.186446 |
| ENSG00000021826 | 3.517551 | 9.387474 | 4.644857 | 1.05E-05 | 0.000268 | 3.109274 |
| ENSG00000137699 | 2.340764 | 9.269883 | 4.552004 | 1.52E-05 | 0.000354 | 2.763356 |
| ENSG00000204305 | -2.00015 | 10.16988 | -4.40211 | 2.72E-05 | 0.000555 | 2.214521 |
| ENSG00000117983 | 3.127142 | 12.21617 | 4.316927 | 3.76E-05 | 0.000715 | 1.90805 |
| ENSG00000153446 | -2.57765 | 11.18359 | -4.31069 | 3.85E-05 | 0.000728 | 1.885786 |
| ENSG00000165376 | -2.27149 | 7.957435 | -4.28121 | 4.31E-05 | 0.000794 | 1.780753 |
| ENSG00000128422 | 2.120063 | 10.73512 | 4.222627 | 5.38E-05 | 0.000945 | 1.573512 |
| ENSG00000171885 | -2.33519 | 10.11803 | -4.06269 | 9.74E-05 | 0.001498 | 1.017819 |
| ENSG00000168484 | -3.53144 | 12.2324 | -4.018 | 0.000115 | 0.001687 | 0.865228 |
| ENSG00000171564 | 3.353238 | 7.873026 | 3.995512 | 0.000124 | 0.001796 | 0.788922 |
| ENSG00000160182 | 2.608998 | 7.161368 | 3.992447 | 0.000126 | 0.00181 | 0.778546 |
| ENSG00000171560 | 3.505123 | 10.06274 | 3.973626 | 0.000135 | 0.001904 | 0.71494 |
| ENSG00000187908 | -2.42143 | 11.16713 | -3.92088 | 0.000163 | 0.002205 | 0.537853 |
| ENSG00000161798 | -2.27533 | 8.559755 | -3.87787 | 0.00019 | 0.002488 | 0.394679 |
| ENSG00000101210 | 2.347207 | 9.244942 | 3.875124 | 0.000192 | 0.002506 | 0.385594 |
| ENSG00000161055 | -2.56144 | 10.39325 | -3.76633 | 0.000282 | 0.003365 | 0.02888 |
| ENSG00000162896 | -2.31576 | 12.78273 | -3.72302 | 0.000328 | 0.003786 | -0.11105 |
| ENSG00000215182 | 2.798983 | 8.610219 | 3.705831 | 0.000348 | 0.003973 | -0.16623 |
| ENSG00000104760 | 2.062843 | 7.57742 | 3.209128 | 0.001796 | 0.013897 | -1.67536 |
| ENSG00000130294 | -2.07062 | 7.779121 | -3.19983 | 0.001849 | 0.01418 | -1.70197 |
| ENSG00000171557 | 2.65092 | 10.81373 | 3.186409 | 0.001929 | 0.014628 | -1.74027 |
| ENSG00000151632 | 2.267472 | 11.07773 | 2.971499 | 0.003722 | 0.024185 | -2.3356 |
| ENSG00000096088 | -2.03425 | 10.11781 | -2.71789 | 0.00776 | 0.042147 | -2.99328 |

**Supplementary Table 4**

|  | logFC | AveExpr | t | P.Value | adj.P.Val | B |
| --- | --- | --- | --- | --- | --- | --- |
| ZNF385B | -3.5072 | 6.223957 | -4.48943 | 0.000152 | 0.014618 | 1.004733 |
| HSD17B6 | -3.03031 | 7.983271 | -6.66976 | 6.72E-07 | 0.002181 | 6.005243 |
| APOH | -2.96621 | 6.260801 | -3.82277 | 0.000823 | 0.032786 | -0.55814 |
| SUSD2 | -2.96443 | 8.135912 | -4.41844 | 0.000182 | 0.016034 | 0.837307 |
| ADH1B | -2.82817 | 8.366762 | -3.99684 | 0.000531 | 0.026614 | -0.15306 |
| PLA2G1B | -2.7869 | 5.982491 | -4.30354 | 0.000244 | 0.018711 | 0.566563 |
| CYP4B1 | -2.64395 | 7.451238 | -3.98231 | 0.000551 | 0.026993 | -0.18698 |
| HLF | -2.4632 | 5.974891 | -4.50817 | 0.000145 | 0.014067 | 1.048944 |
| LRRK2 | -2.41753 | 7.942786 | -3.64686 | 0.001279 | 0.041259 | -0.96386 |
| CHRDL1 | -2.30371 | 7.735639 | -3.89241 | 0.000691 | 0.030029 | -0.39644 |
| AQP4 | -2.28356 | 4.995689 | -4.37792 | 0.000202 | 0.017047 | 0.741778 |
| BEX5 | -2.22486 | 7.140637 | -4.98415 | 4.32E-05 | 0.008352 | 2.170636 |
| MIR29B2CHG | -2.13306 | 6.748487 | -4.04116 | 0.000475 | 0.024987 | -0.04945 |
| CLIC5 | -2.07656 | 6.791658 | -3.8465 | 0.000776 | 0.031694 | -0.50311 |
| ACADL | -2.04539 | 5.170785 | -4.29652 | 0.000248 | 0.018865 | 0.550046 |
| FREM2 | -1.99789 | 5.066204 | -4.21415 | 0.000306 | 0.020586 | 0.356272 |
| FGFR2 | -1.9844 | 7.269489 | -4.91237 | 5.19E-05 | 0.00887 | 2.001872 |
| ATP13A4 | -1.96363 | 5.532273 | -3.76235 | 0.000958 | 0.035478 | -0.69797 |
| TMED6 | -1.91048 | 4.996513 | -3.53112 | 0.001705 | 0.047326 | -1.22831 |
| TNIK | -1.87493 | 5.798069 | -5.60853 | 8.98E-06 | 0.004157 | 3.625129 |
| P3H2 | -1.84497 | 7.218063 | -3.89431 | 0.000688 | 0.030011 | -0.39203 |
| ANKRD29 | -1.83424 | 4.827566 | -4.89968 | 5.36E-05 | 0.008908 | 1.972001 |
| CACHD1 | -1.77801 | 6.69633 | -3.53798 | 0.001676 | 0.046945 | -1.2127 |
| MMP28 | -1.75787 | 5.156962 | -3.56828 | 0.001555 | 0.045128 | -1.14366 |
| METTL7A | -1.70247 | 10.08218 | -5.25504 | 2.18E-05 | 0.006353 | 2.80513 |
| RNASE1 | -1.67166 | 11.41242 | -3.73164 | 0.001035 | 0.036781 | -0.76885 |
| CLIC3 | -1.62075 | 7.087731 | -3.93036 | 0.000628 | 0.028853 | -0.30813 |
| IL33 | -1.61145 | 6.452657 | -3.80656 | 0.000857 | 0.033544 | -0.59571 |
| ZMAT1 | -1.60563 | 5.167032 | -3.68138 | 0.001173 | 0.039434 | -0.8846 |
| FCN3 | -1.59829 | 6.922481 | -3.67683 | 0.001187 | 0.039707 | -0.89505 |
| CBX7 | -1.59748 | 7.407482 | -6.20949 | 2.04E-06 | 0.0025 | 4.990222 |
| ZNF204P | -1.57323 | 6.450878 | -4.14333 | 0.000366 | 0.022155 | 0.189934 |
| FMO2 | -1.5353 | 7.120677 | -3.88728 | 0.0007 | 0.03015 | -0.40838 |
| CAPN3 | -1.53276 | 6.275608 | -3.77866 | 0.00092 | 0.03482 | -0.66026 |
| IL6R | -1.52107 | 7.065008 | -5.03998 | 3.75E-05 | 0.007602 | 2.301733 |
| CEBPA | -1.51636 | 7.92269 | -4.23428 | 0.000291 | 0.02042 | 0.403594 |
| FOSL1 | 1.500622 | 5.360305 | 4.119063 | 0.00039 | 0.02258 | 0.133015 |
| TNFAIP6 | 1.525119 | 6.463796 | 3.542266 | 0.001659 | 0.046746 | -1.20294 |
| CDK5R1 | 1.532095 | 4.670629 | 5.457199 | 1.31E-05 | 0.005085 | 3.275381 |
| PITX1 | 1.534855 | 5.565464 | 4.481566 | 0.000155 | 0.014845 | 0.986186 |
| NAMPT | 1.551761 | 8.398909 | 5.087694 | 3.33E-05 | 0.007471 | 2.413664 |
| RHOV | 1.567405 | 6.171414 | 5.695013 | 7.24E-06 | 0.003872 | 3.824016 |
| FA2H | 1.601792 | 6.991847 | 3.689474 | 0.00115 | 0.039257 | -0.86598 |
| SLC39A4 | 1.621684 | 8.352162 | 6.099703 | 2.66E-06 | 0.002779 | 4.743995 |
| HMGB3P1 | 1.623563 | 7.071127 | 4.461987 | 0.000163 | 0.015012 | 0.940004 |
| CDCA5 | 1.629292 | 6.547109 | 4.47381 | 0.000158 | 0.014911 | 0.96789 |
| KRT19 | 1.631786 | 10.74924 | 4.232653 | 0.000292 | 0.02042 | 0.399769 |
| KIF18B | 1.633964 | 6.685371 | 4.310203 | 0.00024 | 0.018499 | 0.582257 |
| GINS1 | 1.637072 | 6.784128 | 3.652828 | 0.00126 | 0.040941 | -0.95018 |
| ZWINT | 1.641516 | 8.20353 | 3.859469 | 0.000751 | 0.031289 | -0.473 |
| UBE2T | 1.643599 | 7.567756 | 3.990564 | 0.000539 | 0.026728 | -0.16771 |
| SLC7A5 | 1.647216 | 7.529477 | 4.544071 | 0.000132 | 0.013273 | 1.133645 |
| KRT8 | 1.659623 | 10.72729 | 5.236483 | 2.28E-05 | 0.006353 | 2.761817 |
| HHIPL2 | 1.676858 | 5.184715 | 3.806264 | 0.000858 | 0.033544 | -0.59639 |
| HJURP | 1.687443 | 6.238167 | 4.07544 | 0.000435 | 0.023648 | 0.030786 |
| F3 | 1.705984 | 8.844579 | 4.137666 | 0.000372 | 0.022155 | 0.17665 |
| CHEK1 | 1.708381 | 5.124329 | 4.333115 | 0.000226 | 0.018088 | 0.636219 |
| SLC16A3 | 1.733765 | 7.939482 | 5.169702 | 2.70E-05 | 0.006795 | 2.605727 |
| FSCN1 | 1.741169 | 6.869804 | 4.708489 | 8.71E-05 | 0.01101 | 1.521532 |
| FOXM1 | 1.744852 | 6.300893 | 3.787628 | 0.000899 | 0.034357 | -0.63952 |
| TK1 | 1.753084 | 7.844462 | 4.270054 | 0.000266 | 0.019626 | 0.487747 |
| KCNMB4 | 1.754101 | 5.949562 | 6.103959 | 2.64E-06 | 0.002779 | 4.753568 |
| MB | 1.769104 | 6.433879 | 3.70343 | 0.00111 | 0.038542 | -0.83386 |
| BUB1B | 1.778896 | 6.919321 | 3.490246 | 0.001887 | 0.04988 | -1.32114 |
| MCM10 | 1.780586 | 5.46398 | 3.805154 | 0.00086 | 0.033554 | -0.59896 |
| KIF14 | 1.783116 | 6.418671 | 3.678779 | 0.001181 | 0.039607 | -0.89057 |
| STEAP1 | 1.800264 | 8.060228 | 3.795605 | 0.000881 | 0.034113 | -0.62107 |
| DSP | 1.837128 | 10.61839 | 4.892538 | 5.46E-05 | 0.008908 | 1.955195 |
| AURKB | 1.844725 | 5.774406 | 3.894627 | 0.000687 | 0.030011 | -0.3913 |
| CENPU | 1.846108 | 7.78128 | 4.399469 | 0.000191 | 0.016479 | 0.792584 |
| UCK2 | 1.878298 | 7.321157 | 4.658541 | 9.89E-05 | 0.011821 | 1.40372 |
| KIF4A | 1.896503 | 6.231345 | 3.579128 | 0.001514 | 0.044593 | -1.11889 |
| SMKR1 | 1.917905 | 5.051831 | 4.350546 | 0.000216 | 0.017711 | 0.677281 |
| CPE | 1.926866 | 9.201221 | 4.200727 | 0.000317 | 0.020586 | 0.324723 |
| MAD2L1 | 1.950577 | 6.535168 | 3.85463 | 0.00076 | 0.031355 | -0.48424 |
| CTHRC1 | 1.952925 | 10.44736 | 3.761715 | 0.00096 | 0.035478 | -0.69944 |
| TRIP13 | 1.954161 | 7.165608 | 4.233176 | 0.000292 | 0.02042 | 0.400998 |
| CCNA2 | 1.988187 | 5.825039 | 3.889038 | 0.000697 | 0.03015 | -0.4043 |
| TPX2 | 2.012906 | 7.184408 | 3.609081 | 0.001405 | 0.043041 | -1.05043 |
| HMGB3 | 2.034403 | 9.449466 | 4.665532 | 9.71E-05 | 0.011782 | 1.42021 |
| CDKN3 | 2.0515 | 6.828897 | 3.612964 | 0.001391 | 0.042741 | -1.04154 |
| S100A9 | 2.066541 | 9.724734 | 3.68991 | 0.001148 | 0.039257 | -0.86498 |
| CDC20 | 2.072122 | 6.975562 | 3.530939 | 0.001706 | 0.047326 | -1.22872 |
| CDC6 | 2.076033 | 5.643449 | 4.334556 | 0.000225 | 0.018088 | 0.639613 |
| KISS1R | 2.095702 | 6.365852 | 3.681169 | 0.001174 | 0.039434 | -0.88508 |
| NUF2 | 2.102877 | 6.026033 | 3.758567 | 0.000967 | 0.035586 | -0.70671 |
| FHL2 | 2.114266 | 8.920521 | 4.577755 | 0.000121 | 0.01289 | 1.213122 |
| MELK | 2.124798 | 7.487447 | 3.830952 | 0.000806 | 0.032345 | -0.53918 |
| CENPA | 2.139931 | 6.616565 | 3.995215 | 0.000533 | 0.026614 | -0.15684 |
| SCIN | 2.171651 | 6.260947 | 3.768343 | 0.000944 | 0.035158 | -0.68412 |
| KRT80 | 2.183869 | 6.722566 | 5.129916 | 2.99E-05 | 0.007168 | 2.512598 |
| RRM2 | 2.186245 | 8.525707 | 3.640099 | 0.001301 | 0.041446 | -0.97938 |
| GJB2 | 2.1917 | 7.430823 | 3.514084 | 0.001779 | 0.048379 | -1.26704 |
| CEP55 | 2.205101 | 6.610619 | 3.863284 | 0.000744 | 0.031084 | -0.46415 |
| DKK1 | 2.259413 | 5.24645 | 3.650287 | 0.001268 | 0.041137 | -0.95601 |
| UHRF1 | 2.298684 | 7.480961 | 5.149939 | 2.84E-05 | 0.007058 | 2.55948 |
| NEK2 | 2.333697 | 6.367865 | 3.879034 | 0.000715 | 0.030425 | -0.42755 |
| MUC16 | 2.359408 | 5.4834 | 5.078557 | 3.40E-05 | 0.007471 | 2.392242 |
| ANLN | 2.363885 | 6.327064 | 3.992029 | 0.000537 | 0.026693 | -0.16429 |
| UBE2C | 2.414979 | 8.331269 | 3.934107 | 0.000622 | 0.028684 | -0.29939 |
| PBK | 2.467486 | 6.558893 | 3.848629 | 0.000771 | 0.03168 | -0.49817 |
| KRT7 | 2.49188 | 9.929189 | 4.828256 | 6.42E-05 | 0.00937 | 1.803838 |
| DUSP4 | 2.642886 | 7.809963 | 4.621576 | 0.000109 | 0.012389 | 1.316513 |
| CYP24A1 | 2.891716 | 5.556325 | 4.267287 | 0.000267 | 0.019626 | 0.481237 |
| SLC16A14 | 2.985996 | 7.957735 | 4.587134 | 0.000119 | 0.012686 | 1.23525 |
| MMP1 | 3.218919 | 7.478566 | 4.087684 | 0.000422 | 0.023162 | 0.059465 |
| S100P | 3.306936 | 9.798964 | 3.601498 | 0.001432 | 0.043352 | -1.06777 |
| FAM83A | 4.976712 | 6.709143 | 25.64563 | 5.89E-19 | 1.23E-14 | 26.30686 |
