# Supplementary figures and images for: High-risk histological subtype-related FAM83A hijacked FOXM1 transcriptional regulation to promote malignant progression in lung adenocarcinoma
Source: PeerJ. 2023 Oct 26;11:e16306. doi: 10.7717/peerj.16306 (PMC10613442; doi:10.7717/peerj.16306)

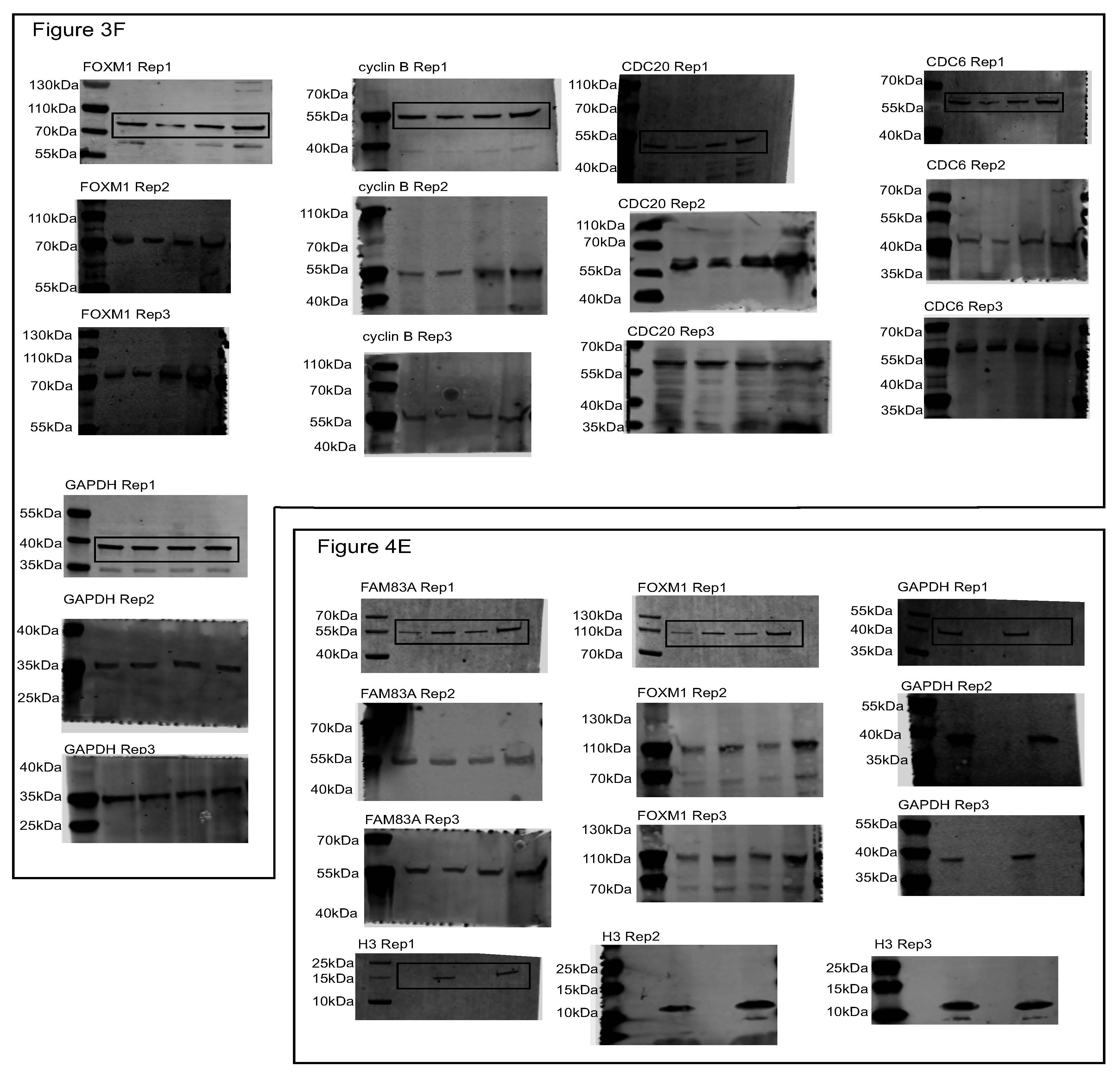

Supplement: Supplemental Information 2 [file peerj-11-16306-s002.jpg]
